# Reciprocal Influence of Chronic Stress on Depressive Symptoms Among Middle-Aged and Older Couples

**DOI:** 10.1093/geroni/igaf122.3305

**Published:** 2025-12-31

**Authors:** Kyuho Lee, Jaemin Jeon, Wi Hoon Jung

**Affiliations:** Daegu University, Gyeongsan, Kyongsang-bukto, Republic of Korea; Daegu University, Gyeongsan, Kyongsang-bukto, Republic of Korea; Gachon University, Seongnam, Kyonggi-do, Republic of Korea

## Abstract

**Background:**

Chronic stress in later life is a known risk factor for depression, potentially magnified within spousal dyads as older adults grapple with financial and social challenges. While many studies examine individual predictors of depressive symptoms, fewer focus on how one partner’s stress may spill over onto the other’s mental health. This study investigated the reciprocal effects of husbands’ and wives’ chronic stress on each other’s depressive symptoms among older couples.

**Method:**

We analyzed data from the 2016 and 2020 waves of the Health and Retirement Study (HRS), encompassing 808 married couples aged 50 or older. A two-wave cross-lagged Actor-Partner Interdependence Model (APIM) tested actor (own T1 stress → own T2 depression) and partner (T1 stress → spouse’s T2 depressive symptoms) effects, while controlling for T1 depressive symptoms. This design captures both individual continuity of depression and cross-spousal influences of stress over time.

**Results:**

Depressive symptoms remained stable for husbands and wives. Husbands’ T1 stress did not predict their own T2 depressive symptoms, whereas wives’ T1 stress did. Both husbands’ and wives’ T1 stress predicted spouses’ T2 depressive symptoms, revealing robust cross-spousal “spillover.” Wives’ stress had a stronger impact on husbands’ subsequent depression than vice versa.

**Discussion:**

These findings underscore the dyadic nature of chronic stress in later life, with notable gender differences. A couple-based perspective for depression interventions is warranted, recognizing each partner’s stress can shape the other’s mental health.

